# Deep phenotypic characterization of NY-ESO TCR engineered T cells and tumor in patients with advanced myeloma

**DOI:** 10.1186/2051-1426-3-S2-P295

**Published:** 2015-11-04

**Authors:** Eduardo Davila, Luca Melchiori, Ryan Wong, Gwendolyn Binder-Scholl, Rafael Amado, Bent Jakobsen, Aaron Rapoport

**Affiliations:** 1 University of MD, Baltimore, MD, USA; 2 Adaptimmune, Abingdon, UK; 3 Adaptimmune, Philadelphia, PA, USA

## 

It is now well established that human tumors express unique antigens, however tumor immune evasion mechanisms often prevent effective naturally occurring anti-tumor immune responses. We hypothesized that infusion of T cells that have been genetically modified to express affinity-optimized antigen-specific TCRs may help overcome these barriers. We initiated a study to evaluate the antitumor efficacy of T cells engineered with an affinity-enhanced TCR specific for the NY-ESO-1 and LAGE-1 cancer testis antigens (NY-ESO^c259^-T) in multiple myeloma (MM) patients with antigen-positive tumors in the setting of an autologous stem cell transplant (NCT01352286). Clinical outcomes of this study have been previously published; clinical data demonstrated safety and encouraging rates of clinical responses, although durability could be further improved. Therefore, in order to better understand the correlates of response, resistance, and relapse, we initiated a deep characterization of the engineered T cell and tumor phenotype in clinical study participants, and we report the initial findings from these studies here.

To better understand the immune profile of the T cell product and its fate post infusion, baseline and post infusion PBMCs were analyzed by flow cytometry evaluating memory and exhaustion markers (e.g. CD45RA, CCR7, PD-1) and polyfunctionality/cytotoxicity markers (e.g. IFN-γ and Granzyme B). NY-ESO^c259^-T cells were detected by pentamer staining and their phenotype was correlated with clinical response. Hierarchical cluster analysis was applied to identify trends of expression of surface markers in different patient subsets. Analysis of cytokine production and polyfunctionality reveal how polyfunctional CD8+ and CD4+ NY-ESO^c259^-T cells evolve upon infusion and selectively regulate different cytokines, with patients displaying different degrees of polyfunctionality. Analysis of baseline T cell profile in patients against the final manufactured product revealed an interesting evolution in markers expression, with CD8+ cells acquiring the most diverse phenotype, and CD4+ cells retaining a more central memory phenotype.

Some patients relapsed despite a low level presence of NY-ESO^c259^ T in marrow. To better understand the mechanisms through which tumor cells evade T cell–mediated destruction, we evaluated the surface expression of over 330 proteins on myeloma cells from bone marrow samples using a flow cytometry-based array. The results revealed the upregulation of several immunosuppressive markers, including PDL-1, on myeloma cells associated with poor prognosis and trafficking, as well as immunosuppression following T cell infusion. This analysis is currently being extended to include additional time points allowing changes in these markers to be tracked before and after therapy and, where applicable, at points of relapse.

